# Traditional to Molecular Diagnostic Technologies for Solanaceous Crop Viruses

**DOI:** 10.3390/plants15213324

**Published:** 2026-10-27

**Authors:** Mengting Zou, Xurui Wen, Xin Zhang, Zhanlin Bei

**Affiliations:** School of Biological Science and Engineering, North Minzu University, Yinchuan 750021, China; 15183315240@163.com (M.Z.); 20247671@stu.nmu.edu.cn (X.W.); realpal00147@163.com (Z.B.)

**Keywords:** solanaceous crops, viruses, species identification, distribution characteristics, molecular detection technologies

## Abstract

Viral diseases are the primary biological constraint on sustainable solanaceous crop production worldwide. Their high diversity, complex transmission pathways, and frequent mixed infections pose substantial challenges for precise disease management. This review summarizes virus types and transmission mechanisms in major solanaceous crops (tomato, pepper, potato) and specialty crops (goji berry, eggplant, tobacco), focusing on pathways mediated by *Bemisia tabaci* and aphids, as well as mechanical and seed-borne transmission. We then review detection technologies: traditional methods lack sensitivity and specificity; molecular approaches have established a comprehensive system, in which conventional and multiplex RT-PCR suit bulk screening, RT-qPCR enables sensitive quantification, isothermal amplification (LAMP, RPA) supports rapid on-site diagnosis, and high-throughput sequencing (HTS) is irreplaceable for novel virus discovery. Current challenges include limited knowledge of viruses in specialty crops, insufficient field-ready tools, and weak strategies against recombinant strains. Future efforts should elucidate viral genetic variation and host–virus interactions, develop rapid field-deployable detection methods, and support early warning and integrated management of solanaceous crop viral diseases.

## 1. Introduction

The Solanaceae family represents an indispensable core plant group in global agriculture and human nutrition, encompassing a wide range of staple food crops, vegetables, and economic crops [1]. Among them, tomato (*Solanum lycopersicum*), pepper (*Capsicum annuum*), and potato (*Solanum tuberosum*)—as representative crops—play irreplaceable roles in ensuring food supply, promoting rural economic development, and maintaining food security [2]. China is one of the world’s leading producers of solanaceous crops, including potato, tomato, and pepper, ranking among the top globally in both cultivated area and total production, thereby generating considerable economic returns and social benefits for both domestic and international markets [3].

However, the expansion of intensive cultivation, climate change, and increasing cross-regional trade have made viral diseases the primary biological constraint on the sustainable development of the solanaceous crop industry. These diseases are characterized by diverse transmission routes, rapid viral evolution, and widespread mixed infections in the field. They not only lead to stunted growth, sharp yield declines, and reduced quality but also significantly increase the difficulty of disease management. Among the “Top Ten Most Economically Damaging Plant Viruses” worldwide, six viruses—including tobacco mosaic virus (*Tobamovirus tabaci*, TMV), tomato spotted wilt virus (*Orthotospovirus tomatomaculae*, TSWV), tomato yellow leaf curl virus (*Begomovirus coheni*, TYLCV), potato virus Y (*Potyvirus yituberosi*, PVY), potato virus X (*Potexvirus ecspotati*, PVX), and cucumber mosaic virus *(Cucumovirus CMV*, CMV)—have solanaceous crops as their primary hosts, further underscoring the importance of these diseases [4]. Currently, more than 312, 187, and 30 virus species have been reported globally as capable of infecting tomato, pepper, and eggplant, respectively [5,6,7]. Furthermore, new recombinant strains continue to emerge, and their geographic distribution is constantly expanding, posing a persistent threat to the safe production of solanaceous crops.

More critically, most plant viruses remain latent during early infection and produce no obvious external symptoms, so visual diagnosis alone delays timely intervention and causes the optimal control window to be missed. Therefore, developing highly sensitive and precise detection technologies—integrated with viral identification and epidemiological analysis—is essential for the sustainable management of solanaceous crop viral diseases [8]. Accordingly, this review summarizes the types and epidemiological characteristics of major solanaceous viruses, evaluates current detection technologies (with emphasis on molecular methods), and discusses their application value and future directions for coping with viral variation and mixed infections.

## 2. Transmission Pathways and Epidemic Characteristics of Major Viruses Infecting Solanaceous Crops

**Figure 1:**
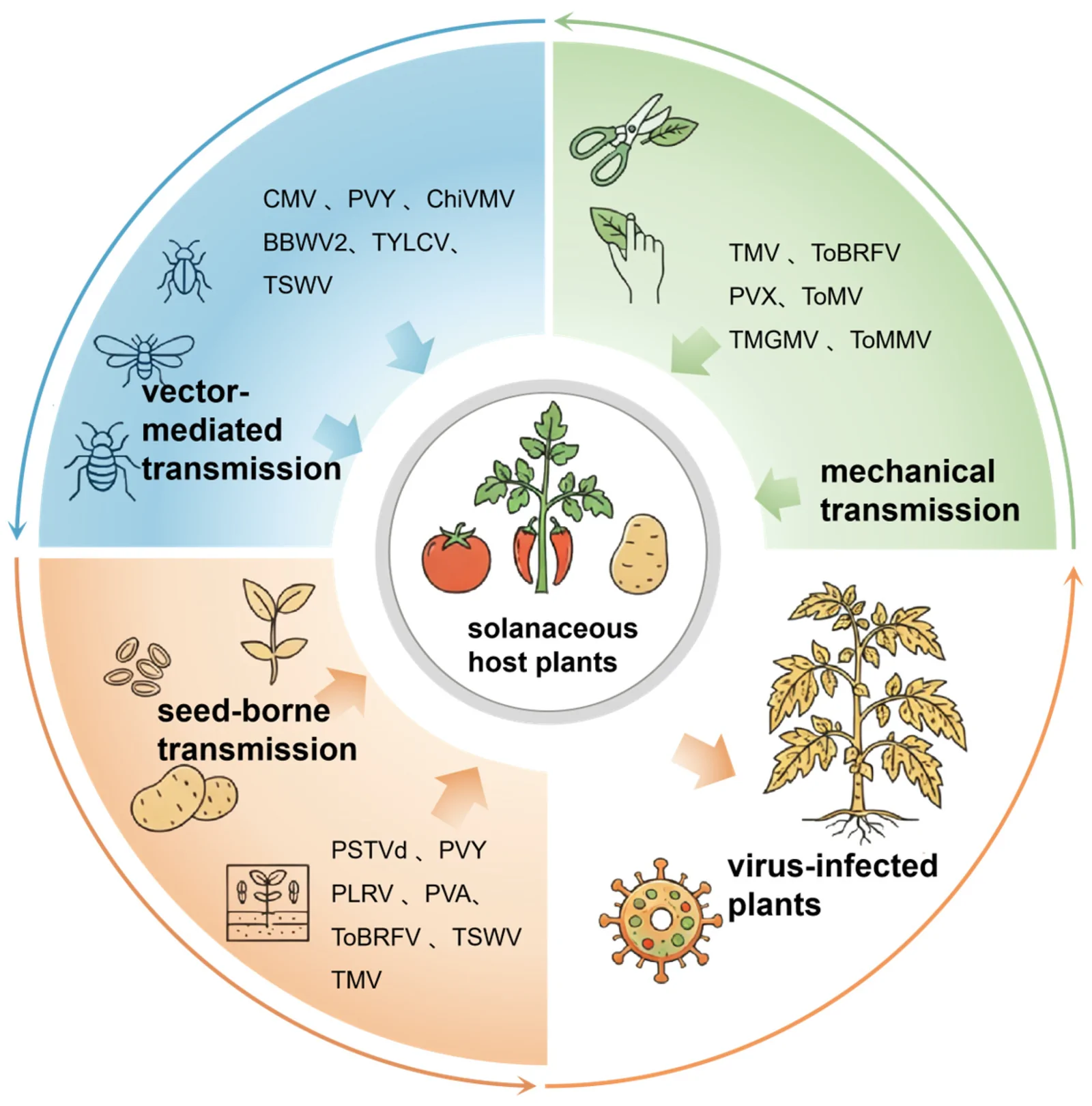
Major transmission pathways of viruses infecting solanaceous crop plants.

Solanaceous crop viruses differ markedly in vector specificity and transmission stability, and their epidemiological traits and damage patterns vary across transmission types. Based on their primary vectors, the major viruses infecting solanaceous crops can be classified into four groups: those transmitted by *Bemisia tabaci*, thrips, aphids, and those spread mechanically or through seed/tubers. These groups differ in host range, geographic distribution, and damage severity, and their diversity collectively underpins the challenges of virus disease management. Figure 1 illustrates the four transmission routes, and the viruses in each group—with their principal hosts, taxonomic family, and key epidemiological features—are summarized in Table 1.

### 2.1. Viruses Transmitted Primarily by Bemisia tabaci and Thrips

Viruses primarily transmitted by whiteflies are among the most destructive pathogen groups in solanaceous crop production, with TYLCV and tomato chlorosis virus (*Crinivirus tomatichlorosis*, ToCV) being the most prominent representatives [9,10]. These viruses are transmitted by *B. tabaci* in either a persistent-circulative (e.g., TYLCV, ToLCNDV) or semi-persistent (e.g., ToCV) manner. They can infect a variety of crops, including tomato and pepper, causing damage across different host species, and represent the dominant viral group in solanaceous crops under protected cultivation.

Originating in the Middle East, TYLCV is now one of the most damaging tomato viruses worldwide, with pandemic distribution across the Mediterranean basin, Africa, the Americas and tropical/subtropical Asia; the Israel (TYLCV-IL) and Mild (TYLCV-Mld) strains are now established in tomato-growing regions on every continent, and global phylogenomic analyses of more than 800 full-length genomes reveal continuous intercontinental spread and recombination-driven diversification [11]. In China, TYLCV is the primary viral pathogen in major protected tomato-growing regions of northern and eastern provinces, with incidence exceeding 60% on protected tomatoes in Shandong, Henan and other areas [12]. It also infects pepper, causing cross-infection damage. Its field prevalence is driven mainly by the migratory spread of *B. tabaci* [13], while interregional transport of infected seedlings is a key route for long-distance transmission. Infection typically causes leaf yellowing and curling, stunted fruit development, and reduced fruit yield and marketability, with per-plant losses of 30–50% [14,15].

First identified in Florida (USA) in the mid-1990s, ToCV is now reported from at least 39 countries across the Americas, the Mediterranean basin, Africa and Asia, with a host range of 119 plant species in 28 families—including tomato, pepper and potato—and field prevalence in tomato that can reach 100% [16]. In China, ToCV infects a broad range of solanaceous crops and is widely distributed in major production areas of northern and southern regions, where co-infection with TYLCV can exceed 40%. Typical symptoms include interveinal chlorosis, leaf thickening and brittleness, and leaf curling, progressing to whole-plant yellowing and premature senescence; mixed infection with TYLCV further exacerbates damage and yield losses [17]. The ToCV + TYLCV mixed infection rate can reach approximately 50% in October, and seedling-stage mixed infections cause particularly severe symptoms and yield losses [18,19].

Tomato leaf curl New Delhi virus (*Begomovirus solanumdelhiense*, ToLCNDV) is another economically important whitefly-transmitted virus that has expanded rapidly beyond its South Asian origin. It is transmitted by *B. tabaci* in a persistent-circulative manner and causes severe leaf curling, yellowing and stunting in tomato and other cucurbit crops [20]. In recent years, ToLCNDV has emerged as a serious threat in the Mediterranean basin and southern Europe, highlighting the risk of further global spread through the international trade in infected planting material [21].

In addition to whiteflies, thrips act as a second important insect vector group for solanaceous viruses. TSWV, the type species of the genus *Orthotospovirus* (family *Tospoviridae*), is transmitted by several thrips species—most notably *Frankliniella occidentalis* and *Thrips tabaci*—in a persistent-propagative manner, meaning that once an adult thrips acquires the virus as a larva, it remains viruliferous for life [22]. TSWV has an extremely broad host range encompassing more than 1000 plant species worldwide, and causes severe spotted wilt, necrotic ringspots and yield losses in tomato, pepper and tobacco. Its rapid global spread over the past century reflects the combined movement of thrips vectors and infected plant material [23].

Collectively, whitefly- and thrips-transmitted viruses—encompassing both semi-persistently and persistently transmitted pathosystems—are favored by hot, dry conditions that promote vector reproduction and by protected cultivation that provides stable overwintering sites, making them the dominant biotic constraint on tomato and pepper production worldwide.

### 2.2. Viruses Transmitted Primarily by Aphids

Aphid-transmitted viruses are the most widely distributed and host-diverse group among solanaceous crops, with key representatives including CMV, PVY, and chilli veinal mottle virus (*Potyvirus capsivenamaculae*, ChiVMV) [24,25]. These viruses all exhibit the typical characteristics of non-persistent aphid transmission, featuring high transmission efficiency and rapid spread. They are commonly found in solanaceous crops such as pepper, potato, and tobacco, and can cause cross-infection between crops, making them a major group involved in complex field infections. The principal aphid vectors involved include the green peach aphid, *Myzus persicae*, the cotton aphid, *Aphis gossypii*, the potato aphid, *Macrosiphum euphorbiae*, and *Neotoxoptera formosana* [26]. These polyphagous species acquire and inoculate viruses during brief probes, and their wide host ranges and rapid reproduction make them highly efficient vectors of non-persistently transmitted potyviruses and cucumoviruses [27,28].

CMV is one of the most widespread plant viruses globally, with an extremely broad host range of over 1200 plant species across more than 100 families [29], and is transmitted in a non-persistent manner by more than 80 aphid species worldwide [30]. Transmitted by aphids, CMV can spread among crops such as pepper, tomato, and tobacco, and often forms complex infections with other viruses, leading to severe plant stunting and leaf deformities, and chlorosis. Infected pepper also exhibits the “three drops” phenomenon—flower drop, fruit drop, and leaf drop—resulting in yield losses of 20–70% [31].

PVY is among the most economically damaging viruses of cultivated potato worldwide and has diversified into multiple strains since its origin in the Andean region of South America [32]. Transmitted non-persistently by over 50 aphid species—chiefly *Myzus persicae* and *Aphis gossypii*—PVY infects pepper, tobacco and other solanaceous crops in addition to potato [33]. In China’s major potato-producing regions, incidence commonly reaches 20–48%, particularly in seed-tuber multiplication systems and continuous-cropping pepper/tobacco fields [34]. PVY induces severe mosaic, leaf drop and vein necrosis in potato; synergistic co-infection with PVX markedly elevates PVX negative-strand RNA accumulation and virion yield, compounding yield losses [35]. In pepper and tobacco, PVY causes systemic necrosis and apical wilt, substantially reducing marketable yield.

Overall, these aphid-transmitted potyviruses and cucumoviruses represent the most widespread and economically significant constraint on solanaceous crop production worldwide; their non-persistent transmission mode, combined with the polyphagous nature and rapid reproductive potential of their aphid vectors, underpins their capacity to cause rapid field epidemics, cross-crop spillover, and complex mixed infections that are difficult to manage through chemical control alone.

### 2.3. Stable Viruses Mainly Transmitted via Mechanical Contact and Seeds

Mechanically and seed-transmitted viruses possess exceptionally stable virions, enabling long-term survival in the field. They can be transmitted through sap via mechanical friction during agricultural operations, or vertically through seeds and tubers. Key representatives include TMV, tomato brown rugose fruit virus (*Tobamovirus fructirugosum*, ToBRFV), and potato spindle tuber viroid *(Pospiviroid fusituberis*, PSTVd) [36]. These environmentally stable viruses persist in the field and are among the most difficult to control in solanaceous crops, causing severe damage to tomato, pepper, potato, and goji berry.

TMV is one of the most widespread and economically damaging tobamoviruses worldwide, with an extremely broad host range encompassing tomato, pepper, tobacco and many other solanaceous and non-solanaceous crops [37]. Its virions are exceptionally stable and can remain infectious for several years in dry plant debris and on inert surfaces. Mechanical friction during agricultural operations such as pruning, topping and harvest is the primary route of field spread, while infected seeds enable long-distance dissemination [38]. TMV causes mottled foliage and fruit in tomato, leaf curling and stunting in pepper, and frequently forms mixed infections with CMV that further exacerbate damage [39]. In the Mediterranean basin and other major protected cropping regions, seed-borne tobamoviruses including TMV and tomato mosaic virus (*Tobamovirus tomatotessellati*, ToMV) remain among the most prevalent viral constraints on pepper and tomato production [40]. In China, TMV prevalence is particularly high in open-field solanaceous-growing regions where agricultural operations are frequent [41].

ToBRFV is an emerging tobamovirus first identified in Jordan in 2015. Since then, it has spread rapidly to over 40 countries across North America, Europe, the Middle East and East Asia, primarily through international trade in infected seeds and seedlings. Its virions are exceptionally stable and highly infectious; mechanical friction during pruning, topping and harvest drives rapid field dissemination, while the high seed transmission rate enables long-distance spread. Critically, ToBRFV overcomes the widely deployed *Tm-*2^2^ resistance gene in tomato, rendering previously resistant cultivars susceptible. Infected fruits develop brown necrotic spots, surface rugosity and irregular ripening, making them unmarketable [42]. ToBRFV now represents one of the most serious emerging threats to protected tomato production worldwide.

Although a viroid rather than a virus, PSTVd shares a similar transmission profile, spreading via infected potato tubers and goji berry seedlings and through mechanical contact during field operations. As a globally regulated quarantine pathogen, PSTVd is routinely included in solanaceous virus-survey panels.

**Figure 2:**
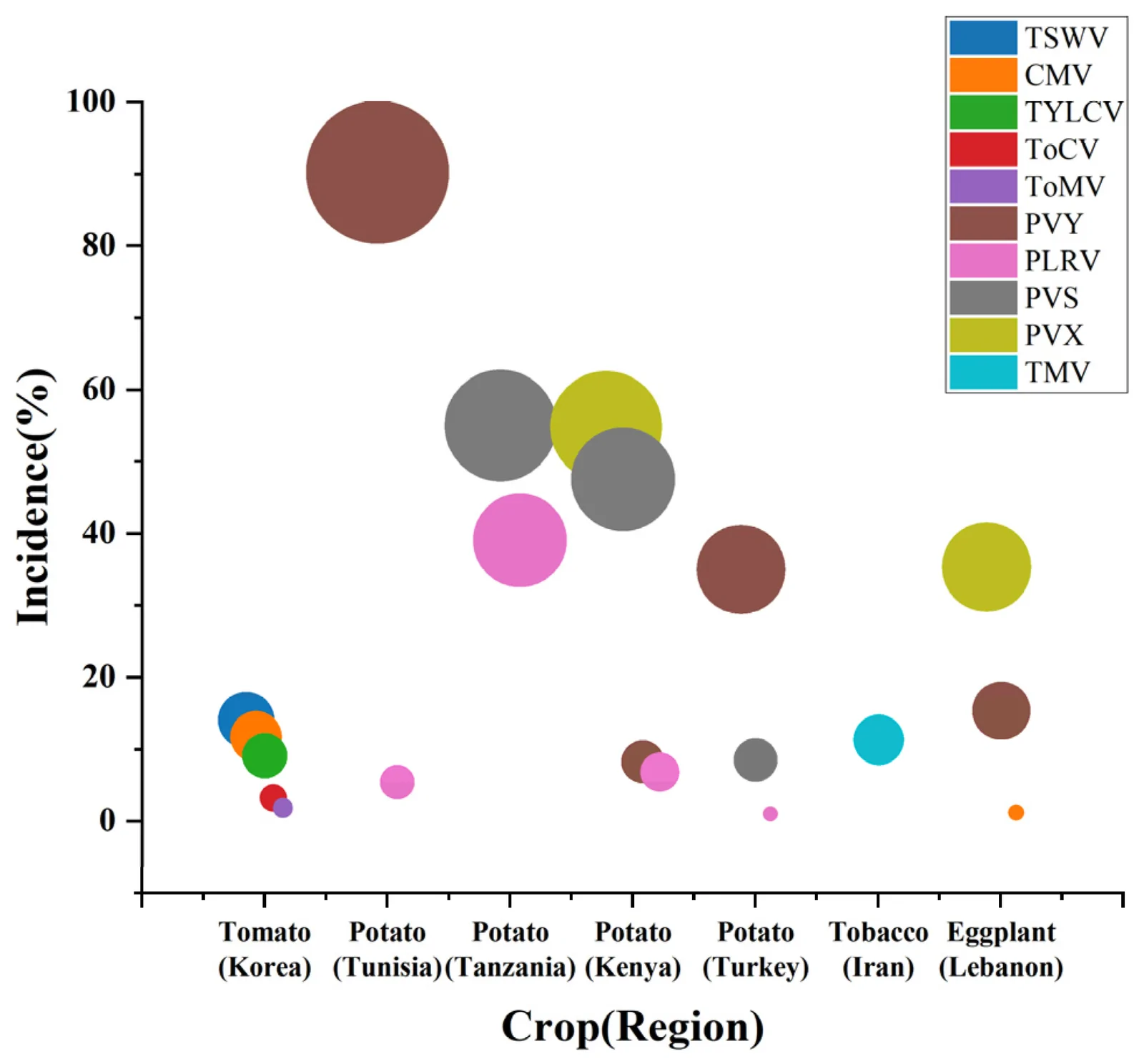
Bubble chart of dominant viral populations in major solanaceous crops. Data were compiled from published field epidemiological surveys conducted in Korea, Tunisia, Tanzania, Kenya, Turkey, Iran, and Lebanon, using detection methods including reverse-transcription PCR (RT-PCR), double antibody sandwich enzyme-linked immunosorbent assay (DAS-ELISA), and serological indexing [43,44,45,46,47,48,49]. Bubble size is proportional to the virus incidence value. Overlapping bubbles within the same crop-region group indicate the co-circulation of multiple viral species in the corresponding host population.

Such mechanically and seed-borne transmitted viruses are not dependent on insect vectors owing to their extraordinary environmental stability, facilitating long-distance spread through infected seedlings and continuous dissemination via field operations. Their persistent presence within host tissues makes them difficult to eradicate, underscoring the importance of seedling testing and hygienic cultivation practices. However, inadequate seedling quarantine at the production level further complicates their management. Figure 2 summarizes the incidence of dominant viruses across major solanaceous crops in different regions of the world.

### 2.4. Challenges for Detection Technologies Posed by Transmission Pathway Complexity

Based on the transmission modes described above, the major solanaceous viruses can be grouped into four categories: whitefly-borne, thrips-borne, aphid-borne, and mechanically/seed-borne. Each group imposes distinct demands on detection technologies.

Whitefly- and thrips-transmitted viruses (e.g., TYLCV, ToCV, TSWV) spread rapidly and cause widespread damage, requiring early detection for timely warning and source containment. Aphid-transmitted viruses (e.g., CMV, PVY, ChiVMV) exhibit extensive strain variation and frequent recombination, making generic assays insufficient; detection tools must be able to discriminate closely related strains. Mechanically and seed/tuber-borne viruses/viroids (e.g., TMV, ToBRFV, PSTVd) are highly stable and spread over long distances via infected planting material, placing high demands on seed and seedling certification. Furthermore, mixed infections involving multiple virus groups are common in the field, requiring multiplex detection capability.

These challenges collectively drive the continuous evolution of solanaceous virus detection from traditional methods toward molecular diagnostics, as discussed in the following section.

## 3. Research Progress on Detection Technologies for Solanaceous Crop Viruses

**Figure 3:**
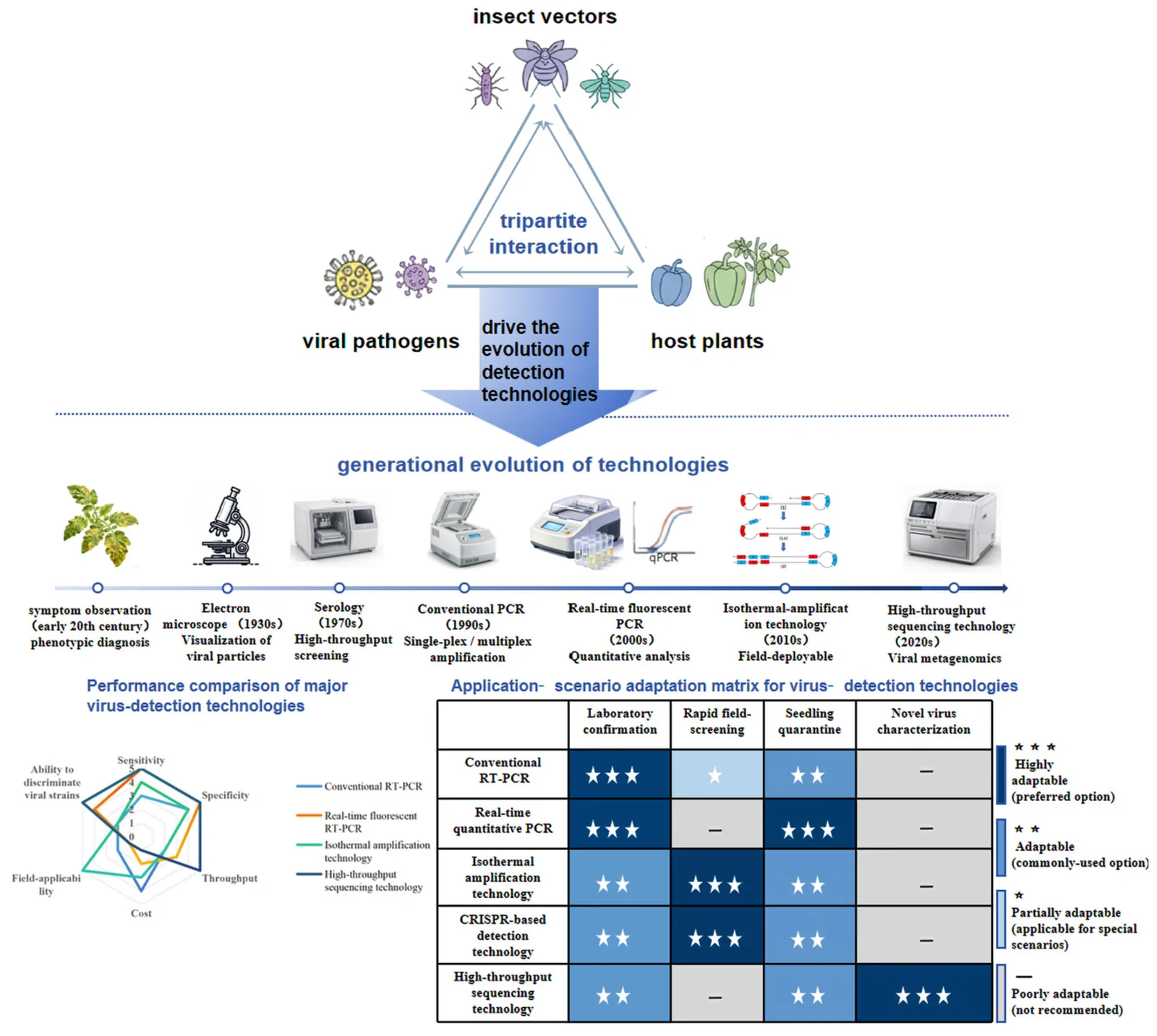
Tripartite interaction of viruses in Solanaceae crops, evolution and application adaptation of detection technologies.

To address the multiple challenges posed by transmission complexity to detection technologies, solanaceous virus detection methods have undergone a generational shift over the past few decades, evolving from phenotypic observation to nucleic acid detection (Figure 3). This section systematically describes this evolutionary process, focusing on the principles and applications of various detection technologies, as well as their advantages and limitations in addressing the aforementioned challenges.

### 3.1. Traditional Detection Technologies

Traditional detection methods for solanaceous viruses fall into two main categories: visual symptom-based identification and classical physicochemical assays [50]. The former category relies on visual diagnosis of typical viral symptoms by experienced plant pathologists, encompassing field symptom inspection and indicator plant bioassays. The latter achieves definitive detection through host-specific inoculation, electron microscopy, and serological assays, leveraging viral infectivity traits, morphological features, or antigenic properties [51].

Biological indexing (indicator plant assay) relies on host-specific symptoms in indicator species such as *Nicotiana benthamiana*, *Chenopodium amaranticolor*, *Gomphrena globosa*, or *Phaseolus vulgaris* [52]. Sap is mechanically inoculated onto young seedlings and maintained in a greenhouse for 1–3 weeks; characteristic local lesions, mosaics, ringspots, or necrosis allow preliminary virus identification. It is specific and inexpensive but labor-intensive, time-consuming, greenhouse-dependent, and unable to detect latent infections.

Serological testing, primarily ELISA, uses virus-specific antibodies to capture coat proteins from plant extracts [8]. In DAS-ELISA, plates are coated with capture antibody, incubated with extract, then with enzyme-conjugated antibody and substrate, and read at 405 nm. ELISA is simple, inexpensive, and automatable for large-scale surveys, but has moderate sensitivity, requires high-quality antibodies, and cannot distinguish closely related strains due to cross-reactivity. Electron microscopy (TEM or immuno-EM) directly visualizes virions and detects low-titer viruses, but requires expensive equipment, skilled personnel, and complex sample preparation, making it unsuitable for large-scale screening [53].

Although traditional detection techniques have improved in operational simplicity and stability, they still suffer from insufficient sensitivity, susceptibility to environmental and host interference, and inability to distinguish closely related strains. These methods—symptom observation, indicator plant assay, electron microscopy, and serological testing (Table 2)—cannot meet modern agriculture’s demands for early detection, precise diagnosis, and strain discrimination, nor can they address rapid viral mutation and mixed infections. Therefore, detection must shift toward higher-resolution methods, and molecular techniques targeting viral genomic nucleotide sequences have become the consensus approach.

### 3.2. Molecular Biological Detection Technologies

Molecular detection technologies targeting viral nucleic acids offer major advantages. By directly analyzing viral genetic material, they exploit sequence specificity and stability to overcome traditional limitations, enabling sensitive detection at early stages and low titers, precise strain genotyping, and accurate analysis of mixed infections. These capabilities provide critical support for closed-loop management of solanaceous viral diseases, from early warning to integrated control.

#### 3.2.1. Conventional RT-PCR

Reverse transcription polymerase chain reaction (RT-PCR) assays are broadly classified into single-plex and multiplex formats based on testing throughput. Single-plex RT-PCR uses one pair of virus-specific primers per reaction to detect a single target and is suitable for precise screening of known viruses. Multiplex RT-PCR was developed to address the widespread mixed infections in field-grown solanaceous crops, enabling simultaneous detection of multiple viruses in a single reaction to simplify operations, shorten testing time and reduce costs [54].

Multiplex RT-PCR has been widely applied for solanaceous virus detection, with progress in system optimization. One optimization, dual-priming oligonucleotide (DPO) primers—with two priming regions linked by polydeoxyinosine—reduces non-specific annealing and mismatched extension. They are less sensitive to annealing temperature, making them useful in multiplex reactions. Yuan et al. (2021) designed DPO primers and established a dual DPO-RT-PCR detection system for the quarantine viruses peanut stunt virus (*Cucumovirus* PSV, PSV) and tomato ringspot virus (*Nepovirus lycopersici*, ToRSV) [55]. This system exhibits high specificity and is insensitive to annealing temperature, making it suitable for rapid, high-throughput screening at ports of entry. Bostan and Peker (2009) investigated the feasibility of using quadruplex RT-PCR to detect potato virus S *(Carlavirus sigmasolani*, PVS), potato leafroll virus (*Polerovirus* PLRV, PLRV), PVX, and PVY in dormant potato tubers; by optimizing reaction conditions, they reliably detected PVS, PLRV, and PVX [56]. Wang et al. (2005) designed specific primers based on the capsid protein (CP) or P1 gene sequences of PVX, PVY, potato virus A (*Potyvirus atuberosi*, PVA), PVS, and PLRV and established a multiplex RT-PCR detection system [57]. This system is over 100 times more sensitive than ELISA and enables simultaneous detection of multiple target viruses in samples with mixed infections. For all RT-PCR amplification formats, amplicon identity is routinely confirmed by bidirectional Sanger sequencing followed by BLASTn alignment (NCBI BLAST+ 2.17.0) against the GenBank database. This verification step validates primer specificity, excludes cross-amplification of closely related viruses or strains, and is particularly valuable for assays targeting highly variable viruses or yielding unexpected band sizes.

In summary, conventional and multiplex RT-PCR provide a robust and widely adopted platform for detecting single and mixed virus infections in solanaceous crops, although their qualitative output limits their utility for viral load monitoring.

#### 3.2.2. Real-Time Quantitative RT-PCR (RT-qPCR)

RT-qPCR is a nucleic acid amplification technique that quantifies the initial template by incorporating fluorescent probes into the PCR reaction system, monitoring the accumulation of fluorescent signals in real time during the amplification process, and establishing a standard curve using standards of known concentrations [58]. This technique is characterized by high specificity, high sensitivity, and ease of operation, and has been widely applied in scientific research and industrial practice. Yang et al. (2024) established corresponding RT-qPCR detection systems for PVY, PVS, and PLRV—three viruses that cause severe damage in major potato-producing regions—which demonstrated high amplification efficiency and specificity [59]. Li et al. (2020) established a PVY RT-qPCR detection system based on the conserved sequence of the PVY coat protein; its sensitivity was 1000-fold higher than that of conventional RT-PCR, and the detection rate of PVY in potato samples from five provinces (Shandong, Qinghai, Guizhou, Heilongjiang, and Hebei) was significantly higher than that of conventional RT-PCR [60]. Globally, RT-qPCR has replaced DAS-ELISA as the reference quantitative assay for PVY and PLRV in certified seed potato programs across the EU, North America and Oceania [61]. Its superior detection of low-titer latent tuber infections improves seed-borne virus screening accuracy and virus elimination efficiency [62].

RT-qPCR offers high specificity, sensitivity, and accurate quantification for seed certification and epidemiological monitoring, but its cost, infrastructure requirements, and dependence on known sequences limit field deployment and detection of novel variants, necessitating further optimization.

#### 3.2.3. Isothermal Amplification Technologies

Isothermal amplification techniques are a well-established class of molecular detection methods that enable nucleic acid amplification at a constant temperature. They allow for the exponential amplification of target sequences without the need for a thermal cycler and have been increasingly applied to the rapid detection of solanaceous viruses. Currently, loop-mediated isothermal amplification (LAMP) and recombinase polymerase amplification (RPA) are the most representative techniques.

**Figure 4:**
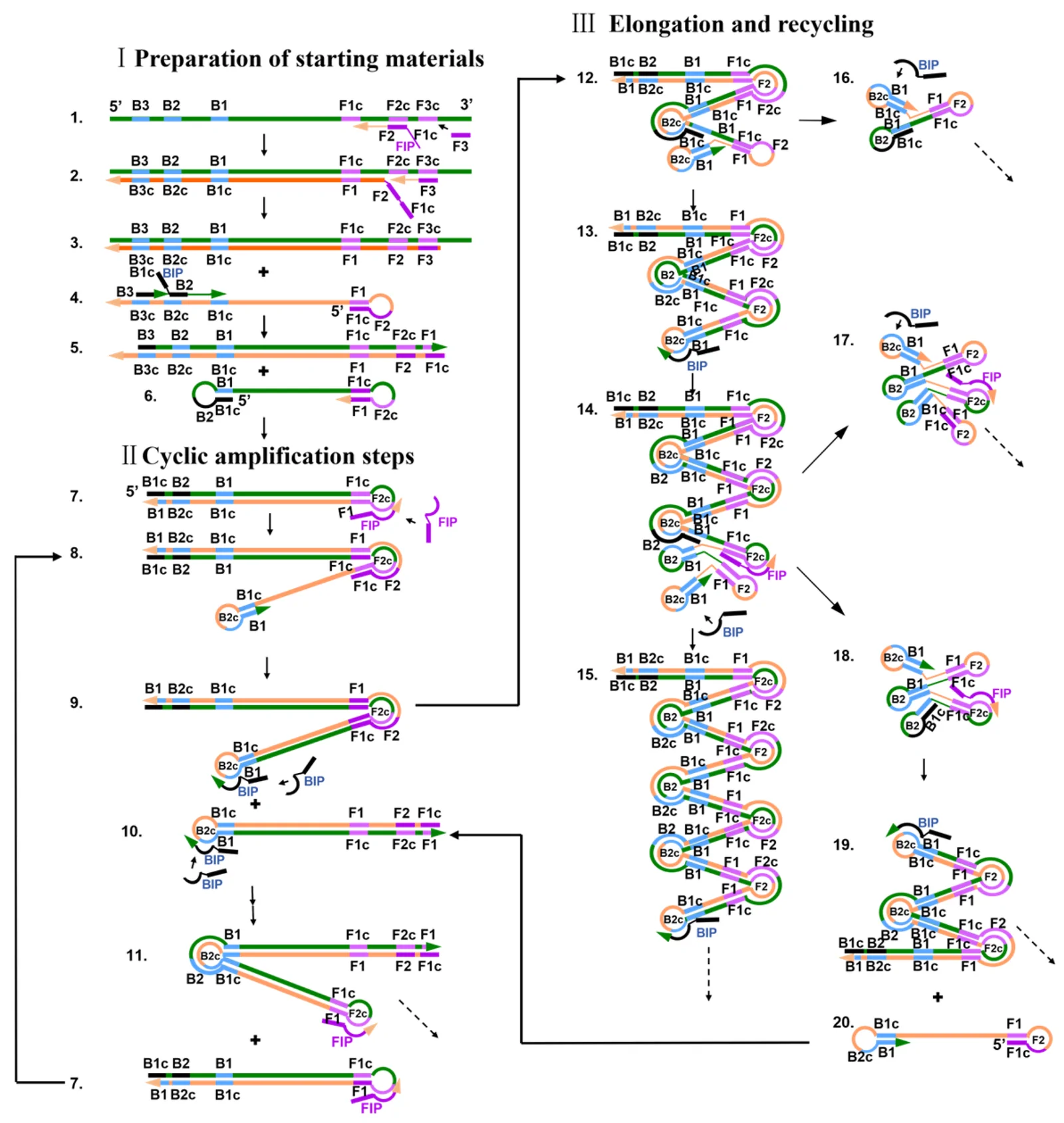
Schematic representation of the LAMP mechanism [63]. Stages I–III cover starting-material production, cycling amplification, and elongation/recycling. Colors distinguish forward-side primers (FIP/F3) from backward-side primers (BIP/B3); solid arrows indicate strand extension and strand-displacement synthesis, and dashed arrows denote the released single-stranded template for the next round.

LAMP is an isothermal nucleic acid amplification technique established by Notomi et al. (2000) (Figure 4) [63]. This technique involves designing four primers targeting six specific regions of the target gene. Under the action of a strand-displacement polymerase, the reaction proceeds at a constant temperature of 60–65 °C, amplifying the DNA template to the million-copy level within 1 h without the need for thermal cycling equipment.

Because LAMP directly amplifies DNA, an additional reverse transcription step is required for RNA viruses, which constitute the majority of solanaceous viruses. To detect RNA viruses prevalent in solanaceous crops, researchers combined the reverse transcription step with LAMP technology to establish a reverse transcription loop-mediated isothermal amplification (RT-LAMP) detection system. Przewodowska et al. (2015) established an RT-LAMP detection method for PVY, which is up to 100 times more sensitive than conventional RT-PCR and shows no cross-reactivity with non-target viruses such as PVS and PLRV [64]. More recent applications have extended RT-LAMP to other important solanaceous viruses. Caruso et al. (2024) developed a real-time RT-LAMP assay for TSWV that was 100-fold more sensitive than RT-qPCR; when applied to field tomato and pepper samples with simplified sample preparation, it raised the positive detection rate to 37% and showed particular advantage in detecting asymptomatic infections [65]. For ToMV, an RT-LAMP assay was developed that could detect the virus in tomato samples within 5 min [66].

**Figure 5:**
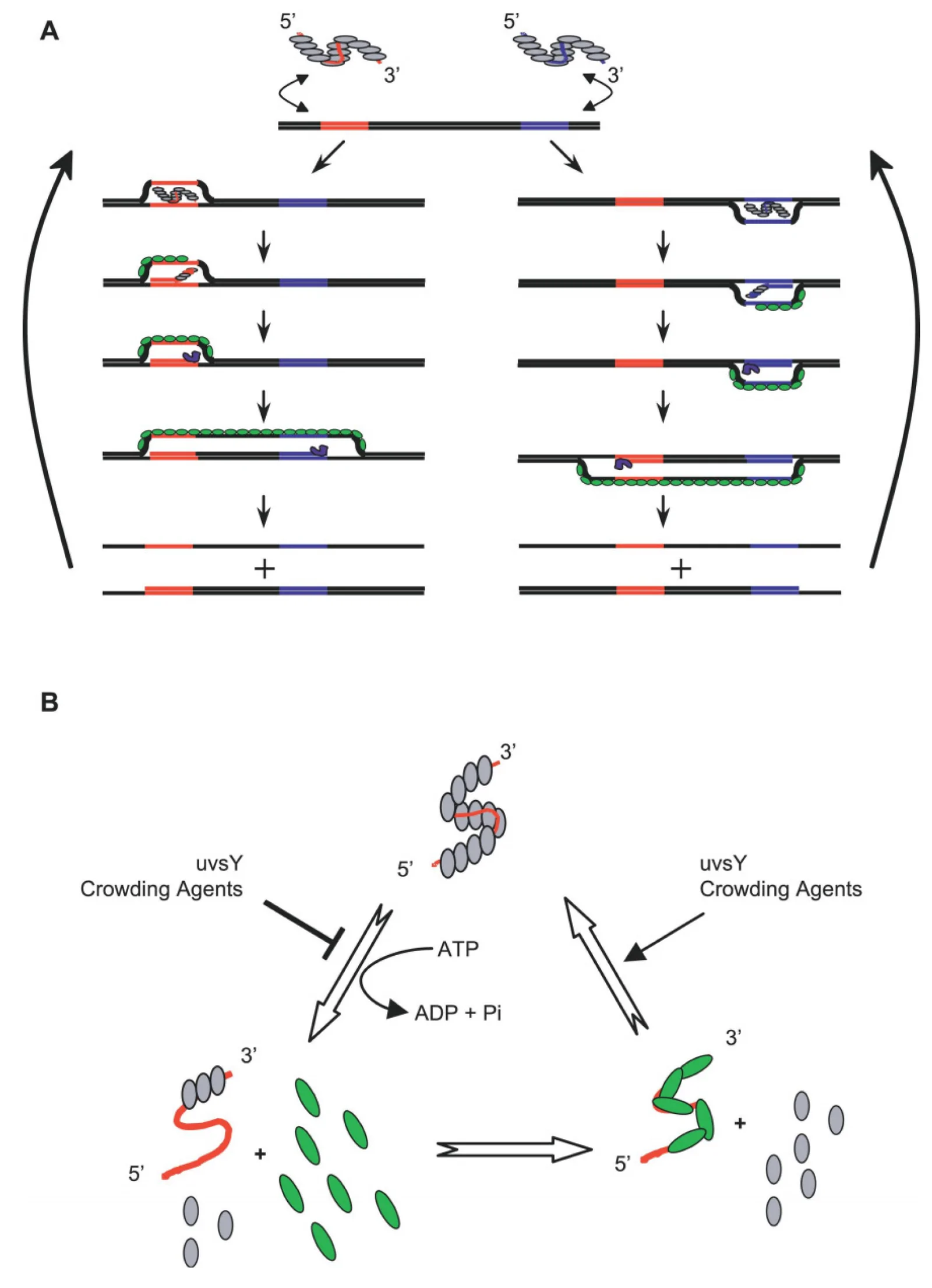
Schematic of RPA reaction mechanism [67]. (**A**) Recombinase–primer filaments scan the template for homologous sequences (red/blue); after strand exchange, the displaced strand is bound by gp32 (green), and primers are extended by Bsu polymerase (blue). (**B**) With ATP, uvsX (gray) loads cooperatively onto primers (red); ATP hydrolysis disassembles the filament and uvsX is replaced by gp32 (green), while uvsY and crowding agents favor recombinase loading. Arrows indicate filament loading, strand exchange, and primer extension.

RPA was first reported by Piepenburg et al. (2006), based on the T4 bacteriophage DNA replication mechanism (Figure 5) [67]. It relies on UvsX recombinase, its cofactor UvsY, single-stranded DNA-binding protein (SSB), and strand-displacing Bsu DNA polymerase. The UvsX–primer complex scans for homologous sequences and invades double-stranded DNA to form a D-loop; SSB stabilizes the displaced strand, and Bsu polymerase extends the new strand, enabling exponential amplification. RPA completes amplification within 20 min at 37–42 °C.

RPA has been widely applied in the detection of pathogens in solanaceous crops. Babu et al. (2018) noted that RPA enables rapid, low-purity amplification at constant temperature and is suitable for solanaceous DNA viruses, including TYLCV [68]. Recent applications have expanded to both RNA and DNA viruses of solanaceous crops. For ToBRFV, an RT-RPA assay coupled with CRISPR-Cas12a and CRISPR-Cas9 enabled visual detection on a lateral flow device within approximately 1 h [69]. A dual RPA assay for ToLCNDV and TYLCV achieved a sensitivity of 10^4^ copies·mL^−1^ in 25 min and could simultaneously detect both viruses in plants and whiteflies [70]. In recent years, the combination of RPA with the CRISPR/Cas system has further enhanced detection performance. Paul et al. (2025) established an RPA-assisted CRISPR-Cas12a (DETECTR) visual detection platform targeting the AC1 gene of chilli leaf curl virus (*Begomovirus chillicapsici*, ChiLCV) [71]. This platform achieved a detection limit as low as 7 fg and could detect the virus directly from crude extracts of chili leaves without the need for complex nucleic acid purification steps.

#### 3.2.4. High-Throughput Sequencing (HTS)

HTS, also referred to as next-generation sequencing (NGS), is a nucleic acid sequencing technology that does not rely on traditional isolation and culture methods or known sequence information [72]. This technology enables unbiased sequencing of all nucleic acids present in a sample and, combined with bioinformatics analysis, facilitates the identification of both known and unknown pathogens. In recent years, it has demonstrated unique advantages in virus identification, virome analysis, and the discovery of new viruses in solanaceous crops.

In the field of solanaceous crop virology, HTS has been widely applied to analyze viral species composition and population structure. Rivarez et al. (2021) noted that HTS technology has identified as many as 312 tomato-associated viruses, satellites, and viroids. Among the 45 viruses newly discovered between 2011 and 2020, 14 were first identified using HTS [5]. In chili pepper virus research, Feng et al. (2017) employed deep small RNA sequencing to analyze chili pepper virus disease samples from the Guiyang region. The results indicated the presence of a mixed infection consisting of broad bean wilt virus 2 (*Fabavirus betaviciae*, BBWV2), ChiVMV, CMV, and bell pepper endornavirus (*Alphaendornavirus capsici*, BPEV) [73]. The detection efficiency of this method was significantly higher than that of traditional detection methods.

HTS offers unique and irreplaceable advantages for novel virus discovery and viral variant surveillance. In vegetatively propagated potato crops, which carry a high risk of viral accumulation, conventional methods fail to adequately resolve latent and mixed infections, whereas HTS enables in-depth dissection of viral population structures and identification of recombinant variants. For high-value specialty crops such as goji berry, HTS has also emerged as a critical identification tool. For instance, small RNA sequencing by Fan et al. (2023) detected multiple viruses, including PSTVd, in Ningxia goji berry samples, laying a foundation for subsequent disease management [74].

Beyond short-read HTS platforms, long-read sequencing from Oxford Nanopore Technologies (ONT) offers a complementary approach for plant virus diagnosis [75]. ONT enables label-free, real-time single-molecule sequencing by measuring ionic current fluctuations as nucleic acid strands translocate through biological nanopores. For solanaceous and specialty crop virology, its core advantages include field portability via the palm-sized MinION device [76], rapid turnaround, and long reads spanning full-length viral genomes, which facilitates de novo assembly of recombinant isolates and discrimination of mixed strains within samples. Recent applications include on-site surveillance of tomato and pepper viruses, direct viral RNA sequencing, and virome profiling of goji berry. The main limitation is a higher raw read error rate relative to short-read platforms, which can be offset by high sequencing depth, consensus correction, and advances in base-calling algorithms. ONT is therefore best positioned as a complement to, rather than a replacement for, established short-read HTS platforms, especially when speed and field deployability are prioritized.

Taken together, the complementary use of short-read and long-read HTS platforms has transformed virome profiling and pathogen discovery in solanaceous and specialty crops, delivering unprecedented resolution of viral population diversity, recombinant variants, and transmission dynamics. These advances not only advance fundamental epidemiological understanding but also underpin the development of next-generation targeted detection assays.

#### 3.2.5. Methods for Strain and Variant Discrimination

Building on the platforms outlined above, targeted approaches have been developed to resolve intra-species diversity, as generic presence/absence assays cannot adequately distinguish vector-transmitted viruses with pronounced strain variation, such as PVY (O, N, and NTN strains), CMV (subgroups IA, II, and III), and recombinant TYLCV variants [77].

PCR-RFLP offers a low-cost method for grouping isolates based on cleavage patterns but is constrained by predefined restriction sites [78]. In solanaceous crops, RT-PCR-RFLP of the coat-protein amplicon has been used to assign CMV isolates from tomato and pepper to subgroups I and II, providing an economical subgroup screen prior to sequencing [79]. Furthermore, strain-specific RT-PCR and TaqMan minor-groove-binding (MGB) probes, targeting divergent genomic regions, enable simultaneous discrimination of major strains in a single sample. For instance, in dormant potato tubers, multiplex RT-PCR targeting the coat-protein gene and recombination junctions can discriminate PVY^O^, PVY^N^, recombinant PVY^N:O^, and PVY^NTN^ isolates, while MGB probes targeting strain-specific SNPs allow closed-tube identification in a single reaction [80].

Although technically demanding, single-strand conformation polymorphism (SSCP) and denaturing gradient gel electrophoresis (DGGE) can reveal sequence variation without prior knowledge. For example, SSCP screening of RT-PCR products grouped 81 field CMV isolates into 14 conformational haplotypes, with representative coat-protein sequences subsequently used to assign subgroups IA/IB; this illustrates how these techniques prioritize isolates for confirmatory sequencing [81].

At a higher resolution, HTS-based variant calling, supported by single-nucleotide polymorphism (SNP) and recombination detection tools, is increasingly deployed to resolve mixed populations and emerging recombinants directly from field samples. For example, in tomato and sweet pepper, HTS resolved co-infecting recombinant PVY strains (N-Wi, NTNa, and NTNb) that conventional assays would score as a single positive; deeper sequencing revealed mixed N- and O-type populations that hampered routine genome assembly [82]. Similarly, large-scale phylogenomic and recombination-detection analyses have tracked the global spread and evolutionary history of TYLCV-IL, TYLCV-Mld, and emerging recombinant variants in tomato populations [83].

Taken together, these strain-discriminating methods directly address the epidemiological challenges posed by broad host ranges and rapid evolutionary diversification, providing the resolution necessary to track viral evolution, delineate transmission pathways, and support strain-targeted disease management.

## 4. Conclusions and Future Perspectives

This review systematically summarizes the epidemiological features of the three major transmission routes of solanaceous viruses and identifies the core demands on detection technologies: early warning, precise strain typing, seedling certification, and mixed-infection resolution. Building on this foundation, this review provides a comprehensive overview of the evolution of detection technologies: conventional RT-PCR established the foundation for molecular detection, RT-qPCR enabled accurate quantification, isothermal amplification facilitated rapid on-site diagnosis, and HTS proved uniquely valuable for novel virus discovery.

**Figure 6:**
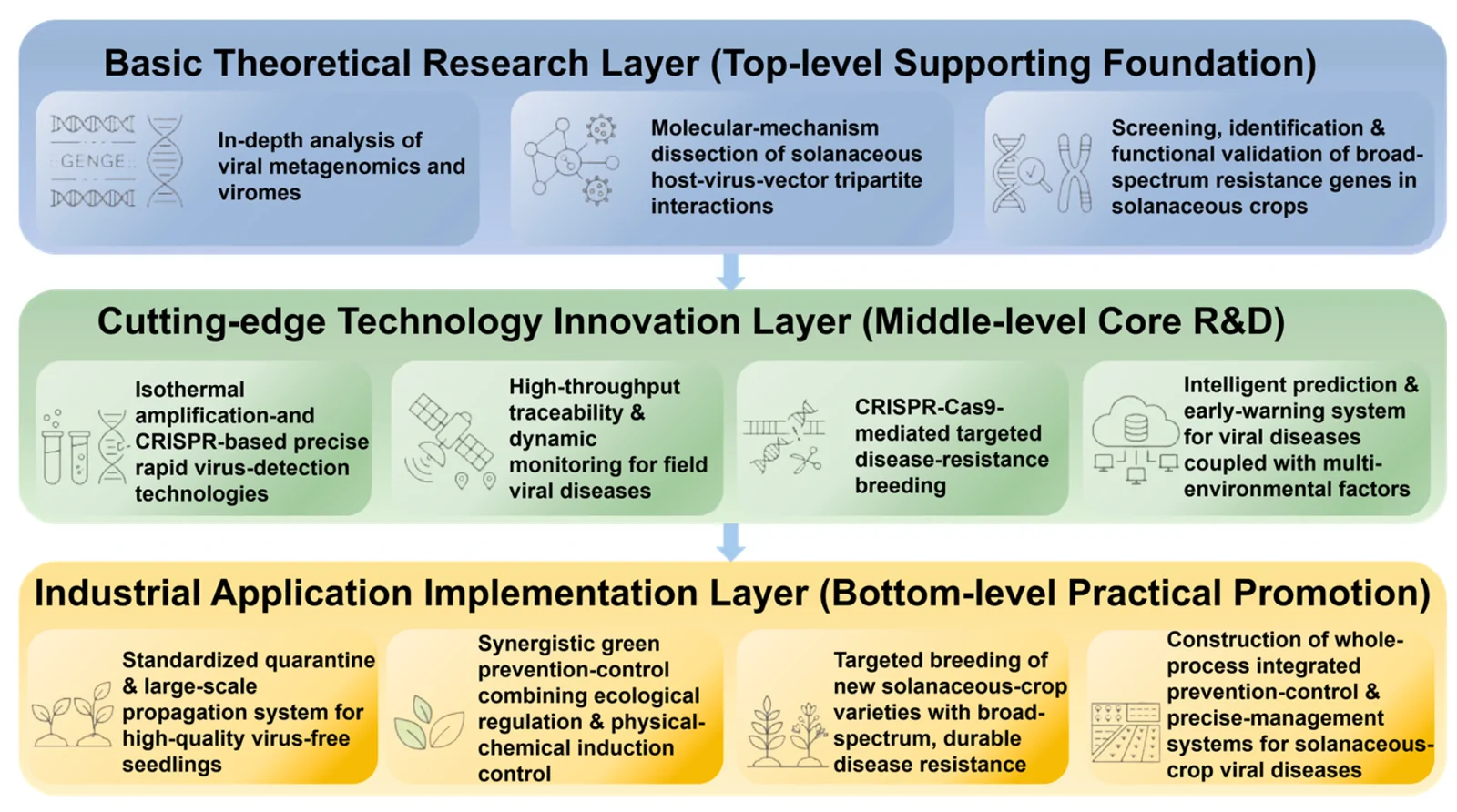
Schematic representation of precise detection and integrated prevention and control of Solanaceae crop viruses: future perspectives.

Despite substantial progress, several critical challenges remain to be addressed. Future efforts should focus on the following areas (Figure 6):

(1) Strengthening fundamental research on viruses infecting specialty crops. Currently, research on viral resource surveys, patterns of genetic variation, and pathogenic mechanisms in specialty crops such as goji berry and eggplant remains relatively weak, with a lack of targeted detection targets and technical systems. On the one hand, efforts must be intensified to conduct systematic surveys of viruses in specialty crops to identify dominant viral populations and their geographic distribution patterns; on the other hand, the interaction mechanisms between viruses and hosts must be thoroughly analyzed to identify highly conserved and specific molecular targets, thereby providing a theoretical foundation for the development of dedicated detection technologies. Based on this, future research will also systematically explore the following directions: First, we will use HTS to construct a comprehensive viral landscape of Ningxia goji berry, identifying dominant viral populations and their geographic distribution characteristics; second, by combining surveys of vector insects with information on seedling transportation, we will analyze the distribution characteristics and transmission patterns of viral communities; third, we will systematically evaluate the impact of viral infection on the active components and visual quality of goji berry to reveal the mechanisms linking viral pathogenicity to fruit quality deterioration.

(2) Advancing field-deployable and multiplex detection technologies. Currently, most molecular detection technologies still rely on laboratory equipment and lack on-site adaptability; furthermore, simultaneous detection systems for mixed infections remain underdeveloped, making it difficult to meet actual field needs. Future research should focus on low-cost, portable, and visual solutions, optimizing coupling schemes between isothermal amplification technology and CRISPR/Cas systems as well as biosensors to develop handheld detection devices. At the same time, efforts should be intensified to develop multiplex detection systems by optimizing primer and probe design and reaction conditions, thereby enabling simultaneous and accurate detection of multiple viruses and enhancing the practicality and efficiency of field applications.

(3) Promoting interdisciplinary integration. Breakthroughs in viral detection technology depend on collaborative innovation across multiple fields. In the future, we must promote interdisciplinary joint research involving molecular biology, bioinformatics, microelectronics, and materials science. On the one hand, this will integrate detection technology with smart agriculture monitoring platforms to enable real-time early warning and dynamic monitoring of viral diseases; on the other hand, it will integrate multidimensional data—such as spectral information, pathological characteristics, and physiological responses—to develop more robust detection models and analytical algorithms. At the same time, we must strengthen the technical integration between seedling quarantine and field prevention and control to establish an integrated “detection–early warning–prevention and control” technical system, providing a solid foundation for the green and sustainable management of viral diseases in solanaceous crops.

## Figures and Tables

**Table 1:** Classification and key characteristics of major viruses infecting solanaceous crops.

Transmission Route	Virus (Abbreviation)	Hosts	Family	Key Characteristics and Impact
Whitefly-borne	Tomato yellow leaf curl virus (TYLCV)	Tomato, pepper	*Geminiviridae*	Incidence >60% in protected tomato; yield loss 30–50%
	Tomato chlorosis virus (ToCV)	Tomato, pepper	*Closteroviridae*	Co-infection with TYLCV > 40%; interveinal chlorosis
Thrips-borne	Tomato spotted wilt orthotospovirus (TSWV)	Tomato, pepper, tobacco	*Tospoviridae*	Worldwide; broad host range; persistent-propagative transmission; severe loss
Aphid-borne	Cucumber mosaic virus (CMV)	Tomato, pepper, tobacco	*Bromoviridae*	Very broad host range; pepper loss 20–70%
	Potato virus Y (PVY)	Potato, pepper, tobacco	*Potyviridae*	Incidence 40–60% in major potato regions
	Chilli veinal mottle virus (ChiVMV)	Pepper, tobacco	*Potyviridae*	Incidence 67.97% in Guizhou tobacco; yield loss >50%
	Broad bean wilt virus 2 (BBWV2)	Pepper, tomato, tobacco	*Secoviridae*	Wilting and necrosis; frequent mixed infections
Mechanical contact	Tobacco mosaic virus (TMV)	Tomato, pepper, tobacco	*Virgaviridae*	Survives decades in dry debris; seed transmission 10–30%
	Tomato brown rugose fruit virus (ToBRFV)	Tomato	*Virgaviridae*	Emerging global threat; fruit rugose and unmarketable
	Potato virus X (PVX)	Potato, tomato, pepper	*Alphaflexiviridae*	Frequently co-infects with PVY, aggravating mosaic and necrosis
	Tomato mosaic virus (ToMV)	Tomato, pepper	*Virgaviridae*	Serologically related to TMV; mosaic and leaf curling
	Tobacco mild green mosaic virus (TMGMV)	Tobacco, tomato	*Virgaviridae*	Mild symptoms; often co-occurs with other viruses
	Tomato mottle mosaic virus (ToMMV)	Tomato, pepper	*Virgaviridae*	First reported 2013; leaf curling and fruit mottling
Seed/tuber-borne	Potato spindle tuber viroid (PSTVd)	Potato, goji berry	*Pospiviroidae*	Detection rate 52.6% in Ningxia goji orchards
	Potato leafroll virus (PLRV)	Potato	*Solemoviridae*	Leaf rolling and stunting; tuber-borne inoculum
	Potato virus S (PVS)	Potato	*Alphaflexiviridae*	Mild or latent symptoms
	Potato virus A (PVA)	Potato	*Potyviridae*	Mosaic and leaf wrinkling

**Table 2:** Comparison of traditional detection technologies for solanaceous crop viruses.

Detection Technology	Principle	Advantages	Disadvantages
Symptom observation	Visual inspection of typical viral symptoms on infected plants	Simple operation; low cost	Low accuracy; affected by cultivar, environment and mixed infections; cannot distinguish viruses with similar symptoms
Indicator plant assay	Sap inoculation onto susceptible indicator hosts; diagnosis based on host-specific symptoms	Relatively specific; low cost	Time-consuming; unsuitable for bulk screening; limited by plant growth conditions; misses latent infections
TEM/ISEM	Transmission electron microscopy (TEM) of virion morphology; immuno-EM (ISEM) improves specificity via antigen–antibody decoration	Direct visualization of virions; detects low-titer viruses	Expensive equipment; complex sample preparation; requires trained personnel; low throughput
Serological assays (e.g., ELISA)	Antigen–antibody reaction based on viral coat proteins	Simple operation; suitable for high-throughput screening; minimal sample preparation	Risk of serological cross-reactivity; time-consuming antibody production; cannot distinguish closely related strains

## Data Availability

No new data were created or analyzed in this study. Data sharing is not applicable to this article.

## Abbreviations

The following abbreviations are used in this manuscript:

TYLCV	Tomato yellow leaf curl virus
ToCV	Tomato chlorosis virus
TSWV	Tomato spotted wilt virus
CMV	Cucumber mosaic virus
PVY	Potato virus Y
PVX	Potato virus X
PVS	Potato virus S
PVA	Potato virus A
PLRV	Potato leafroll virus
PSTVd	Potato spindle tuber viroid
TMV	Tobacco mosaic virus
ToMV	Tomato mosaic virus
ToBRFV	Tomato brown rugose fruit virus
ToMMV	Tomato mottle mosaic virus
TMGMV	Tobacco mild green mosaic virus
ChiVMV	Chilli veinal mottle virus
BBWV2	Broad bean wilt virus 2
BPEV	Bell pepper endornavirus
PSV	Peanut stunt virus
RT-PCR	Reverse Transcription Polymerase Chain Reaction
RT-qPCR	Reverse Transcription Quantitative Polymerase Chain Reaction
LAMP	Loop-Mediated Isothermal Amplification
CRISPR/Cas	Clustered Regularly Interspaced Short Palindromic Repeats/CRISPR-associated protein
HTS	High-Throughput Sequencing
NGS	Next-Generation Sequencing
ELISA	Enzyme-Linked Immunosorbent Assay
ToRSV	Tomato ringspot virus
ChiLCV	Chilli leaf curl virus
ToLCNDV	Tomato leaf curl New Delhi virus
DPO	Dual-priming oligonucleotide
